# Is routine post-sleeve gastrografin needed? Profile of 98 cases

**DOI:** 10.1186/s13104-020-05060-y

**Published:** 2020-04-16

**Authors:** Bandar Saad Assakran, Abdullah Homood Alromaih, Abdulrahman Haitham Alashkar, Fatimah Salem AlGhasham, Mansur Suliman Alqunai

**Affiliations:** 1 Surgical Department, King Fahd Specialist Hospital, P.O. Box 2290, Alnaziyah, Buraidah, 52366 Saudi Arabia; 2 College of Medicine, Sulaiman AlRajhi University, P.O. Box 777, Al Bukayriyah, 51941 Saudi Arabia; 3 College of Medicine, Qassim University, P.O. Box 6655, Almalida, 51452 Saudi Arabia

**Keywords:** Laparoscopic sleeve gastrectomy, Gastrografin, Bariatric surgery, Cost, Hospital stay

## Abstract

**Objective:**

Laparoscopic sleeve gastrectomy (LSG) is one of the most commonly performed bariatric procedures. Some surgeons still perform routine post-sleeve gastrografin (RSG) study believing that it would detect post-LSG complications, especially leak. In this study, we aimed to evaluate the cost-effectiveness of RSG by considering the cost of the study, length of hospital stay and complications-related costs RSG could prevent.

**Results:**

A total of 98 eligible patients were included. Of them, 54 patients underwent RSG and 44 did not. Excluding the cost of LSG procedure, the average cost for those who underwent RSG and those who did not in Saudi Riyal (£) was 5193.15 (1054.77) and 4222.27 (857.58), respectively. The average length of stay (ALOS) was practically the same regardless of whether or not the patient underwent RSG. 90.8% (n = 89) of all patients stayed for 3 days. None of the patients developed postoperative bleeding, stenosis or leak. The mean weight, body mass index (BMI) and percentage weight loss (PWL) 6 months postoperatively were found to be 87.71 kg (SD = 17.51), 33.89 kg/m^2^ (SD = 7.29) and 26.41% (SD = 9.79), respectively. The PWL 6 months postoperatively was 23.99% (SD = 8.47) for females and 30.57 (SD = 10.6) for males (*p *= 0.01).

## Introduction

Bariatric surgery has gained a lot of popularity in fighting obesity as it has proven to be the best long-term solution for this global issue. So, many bariatric procedures have been devised. Although many have considered laparoscopic Roux-en Y gastric bypass (LRYGB) the gold standard bariatric procedure, laparoscopic sleeve gastrectomy (LSG) continues to be the most commonly performed [1]. Compared to other bariatric procedures, such as LRYGB, LSG is equally effective, technically easier to perform and safer [2, 3]. In this procedure, the greater curvature and fundus of the stomach are resected leaving less than 30% of its original volume [4]. This increases the intraluminal pressure of the remaining part of the stomach (i.e. the sleeve), which predisposes to postoperative complications, such as staple line leak [5]. A contrast study that is performed by some surgeons in the first postoperative day for the early detection of such complications is routine post-sleeve gastrografin (RSG) study. It is still performed although several studies have concluded that it is needless considering that such complications are rare and CT scanning is an option that is more sensitive and easier to perform if, for example, leak is clinically suspected, such as when the post-operative patient presents with abdominal pain that is associated with fever and tachycardia or with septic shock [6, 7].

In this study, we reviewed bariatric patients’ data available in the database of the bariatric canter of King Fahd Specialist Hospital (KFSH) in Qassim Region of Saudi Arabia (SA), where RSG is still done for some patients based on the surgeon’s own experience and decision. Our aim was to evaluate the cost-effectiveness of RSG. Fortunately, at least for KFSH bariatric centre and its patients, none of the eligible patients were found to have developed postoperative bleeding, obstruction or leak. So, here we present and discuss the costs and length of hospital stay for the patients who underwent RSG compared to those who did not, and shed light on few observations found when we analysed the patients’ demographic and anthropometric data.

## Main text

### Methods

#### Study design, sitting, eligibility criteria and collected data

This is a cross-sectional study based on the 2018 electronic records of KFSH bariatric patients. Patients were considered eligible if they (1) underwent a LSG at KFSH, (2) had not undergone another bariatric procedure (i.e. LSG was a primary bariatric procedure) and (3) were compliant with their follow-up plan at KFSH for at least 6 months post-operatively.

After we had obtained ethical approval from Al-Qassim Regional Research Ethics Committee, eligible patients were identified and the following data were collected: patients’ demographics; anthropometric measurements of height, immediate preoperative weight and 6 months postoperative weight; length of hospital stay; and total cost excluding the cost of the LSG procedure itself as it is the same for all patients.

#### Operative procedure

The following is how LSG is performed at the bariatric centre of KFSH. The anesthetised patient is placed in reverse Trendelenburg position with slight right lateral tilt. Then, four trocars are used; supra-umbilical, right and left subcostal and epigastric. With the surgeon standing between the patients’ legs, the pylorus is identified and the omentum of the greater curvature is dissected distal to proximal starting 2–3 cm from the pylorus until the gastroesophageal junction and left diaphragmatic crus are identified. After that, the anaesthesiologist introduces a 36 French bougie tube up to the duodenum. Then, the greater curvature and fundus of the stomach are dissected 2–3 cm from the pylorus using endo Gia or power eshlon staplers with 4–5 staples cartilages. Finally, the tube is removed and the staple line is reinforced by reattaching the omentum with v-lock 2/0.

#### Data analysis

Collected data had been corrected for any inconsistencies and then documented in a Microsoft Excel file where the necessary cleaning was done. The final data set was then imported into SPSS software version 23 for windows. The sample’s characteristics were summarised using means, standard deviations (SD) as well as minimum and maximum values for continuous variables and frequencies with percentages for categorical variables. Independent-samples t-test was used for the assessed continuous variables and Chi-square test for the categorical variables with the significance level set at 5%.

### Results

Of the 149 screened patients, 98 were eligible with a mean age of 35 years (SD = 9.92). Of them, 63.3% (n = 62) were females with a mean age of 36 years (SD = 8.94) and 36.7% (n = 36) were males with a mean age of 33.14 years (SD = 11.29). Overall, most patients were 26–35 years of age (n = 39, 39.8%) with the youngest patient operated on being 18 years of age and the oldest being 67. Most female patients (n = 30, 48.4%) were in the age group between 26 and 35 years, while most of the males (n = 13, 36.1%) were between 18 and 25 years of age.

The mean preoperative weight and BMI of the sample were 120.19 kg (SD = 23.4) and 46.54 kg/m^2^ (SD = 11.86), respectively. Females had a statistically significant lower mean preoperative weight (*p *= <0.001) and BMI (*p *= 0.019) compared to males; the mean preoperative weight and BMI for females were 110.08 kg (SD + 16.06) and 44.41 kg/m^2^ (SD = 6.19), respectively; and for males were 137.61 kg (SD = 23.98) and 50.21 kg/m^2^ (SD = 17.36), respectively. Of all 98 patients, 75.51% (n = 74) had a preoperative BMI ≥ 40 kg/m^2^ (i.e. obesity class III) and 24.49% (n = 24) had a preoperative BMI < 40 kg/m^2^.

The mean weight, BMI and percentage weight loss (PWL) 6 months postoperatively were found to be 87.71 kg (SD = 17.51), 33.89 kg/m^2^ (SD = 7.29) and 26.41% (SD = 9.79), respectively. Six months postoperatively, there was no statistically significant difference in mean BMI between females (33.67 kg/m^2^, SD = 5.63) and males (34.28 kg/m^2^, SD = 9.59). However, males lost more weight when expressed in terms of PWL, which was 23.99% (SD = 8.47) for females and 30.57 (SD = 10.6) for males (*p *= 0.01). A summary of demographic and anthropometric characteristics is shown in Table 1, and of a BMI-based categorization in Table 2.

**Table 1:** Comparison of demographic and anthropometric characteristics between males and females pre and postoperatively

Variable	Gender			*p*-value (M vs. F)
	Males (n = 36)	Females (n = 62)	Both (N = 98)	
Mean age in years (± SD) (Minimum/maximum)	33.14 (± 11.29) (18/67)	36.06 (± 8.94) (20/61)	35 (± 9.92) (18/67)	0.16
Age groups (years) n. (%)				
18–25	13 (36.1)	5 (8.1)	18 (18.4)	0.05
26–35	9 (25)	30 (48.4)	39 (39.8)	
36–45	9 (25)	17 (27.4)	26 (26.5)	
> 45	5 (13.9)	10 (16.1)	15 (15.2)	
Mean pre-operative weight in kg (± SD) (Minimum/maximum)	137.61 (± 23.98) (89/198)	110.08 (± 16.06) (80/155)	120.19 (± 23.4) (80/198)	<0.001
Mean pre-operative BMI in kg/m^2^ (± SD) (Minimum/maximum)	50.21 (± 17.36) (34.37/139.2)	44.41 (± 6.19) (29.32/59.8)	46.54 (± 11.86) (29.32/139.2)	0.019
Mean 6 months post-operative weight in kg (± SD) (Minimum/maximum)	95.06 (± 20.23) (63/144)	83.45 (± 14.24) (60/127)	87.71 (± 17.51) (60/144)	0.001
Mean 6 months post-operative BMI in kg/m^2^ (± SD) (Minimum/maximum)	34.28 (± 9.59) (22.32/71.56)	33.67 (± 5.63) (23.44/47.8)	33.89 (± 7.29) (22.32/71.56)	0.7
Percentage weight loss at 6 months post-operatively (± SD) (Minimum/maximum)	30.57 (± 10.6) (6.72/55.09)	23.99 (± 8.47) (3.33/46.49)	26.41 (± 9.79) (33.3/55.09)	0.01

*M* male, *F* female

**Table 2:** Distribution of male and female patients under different BMI categories pre and postoperatively

BMI category	Pre or post-operatively	Males	Females
Normal (%) BMI 18.5–24.9 kg/m^2^	Pre-op	0	0
	Post-op	5 (13.9)	1 (1.6)
Pre-obesity (%) BMI 25–29.9 kg/m^2^	Pre-op	0	1 (1.6)
	Post-op	6 (16.7)	17 (27.4)
Obesity class I (%) BMI 30–34.9 kg/m^2^	Pre-op	2 (5.6)	1 (1.6)
	Post-op	11 (30.6)	23 (37.1)
Obesity class II (%) BMI 35–39.9 kg/m^2^	Pre-op	5 (13.9)	15 (24.2)
	Post-op	7 (19.4)	11 (17.7)
Obesity class III (%) BMI ≥ 40 kg/m^2^	Pre-op	29 (80.6)	45 (72.6)
	Post-op	7 (19.4)	10 (16.1)
Total (%)		36 males (36.7)	62 females (63.3)

*BMI* body mass index

The average overall cost, excluding the cost of the LSG procedure, was 5193.15 Saudi Riyal (1054.77 £) for those who underwent RSG and 4222.27 (857.58 £) for those who did not (p < 0.001). Most patients (90.8%, n = 89) stayed in hospital for 3 days except for 9.2% (n = 9) that stayed for 4 days (n = 4), 5 days (n = 2), 6 days (n = 2) or 10 days (n = 1). Stated differently, the ALOS was practically the same regardless of whether or not the patient underwent RSG. The average total cost and ALOS are summarised in Table 3.

**Table 3:** Average total cost and ALOS for patients who underwent RSG study vs. those who did not

	RSG study		*p*-value
	Underwent (n = 54)	Did not undergo (n = 44)	
Average total in Saudi Riyal (pound sterling)	5193.15 (1054.77)	4222.27 (857.58)	< 0.001
ALOS in days	3.37	3.02	0.05

*ALOS* average length of stay, *RSG* routine post-sleeve gastrografin

All patients where medically free except for 24.49% of the patients (n = 24) who had one or more medical comorbidities of hypothyroidism (n = 16), diabetes mellitus (n = 10), rheumatoid arthritis (n = 3) and/or hypertension (n = 1). Finally, none of the patients developed postoperative bleeding, stenosis or leak.

### Discussion

#### Cost and length of hospital stay

As mentioned previously, the primary goal of our study was to assess the cost-effectiveness of RSG considering its cost, length of hospital stay and the complications-related costs RSG could prevent particularly the most dreaded complication, namely, leak. Luckily, however, all RSG done came back negative and none of the patients developed complications anytime for 6 months postoperatively. Although this zero complications rate at KFSH is quite impressive, it is not unheard of. A systemic analysis published in 2011, which included 4888 patients that had undergone LSG, found the rate of postoperative leak to be as low as 0–7% [8]. In addition, the needlessness and cost-ineffectiveness of RSG was demonstrated in other studies. For example, in a retrospective study published in 2017 and included 200 patients, three of all the gastrografin studies done came back positive and only one was proven to be a true positive [7].

Regarding hospital stay, a typical duration of 3 days was found regardless of whether or not the patient underwent RSG. Nevertheless, is this duration is really needed? Piotr Major et al. assessed in their prospective observational study the risk factors of prolonged hospital stay after LSG. In their study that included 492 patients, their definition of prolonged hospital stay post-LSG was > 3 days. They found that 29.47% of all patients needed a prolonged stay [9]. So, and considering the 9.2% figure found in KFSH bariatric centre, the ALOS at KFSH is not unusual. Still, a 3-day hospital stay is arguably unnecessary and a shorter duration is achievable. Fletcher et al., in a paper published in 2018, reviewed 11,430 patients who had underwent LSG. They found a median length of hospital stay to be 2 days and only 18.4% of patients stayed in hospital for > 2 days [10]. In another study, a yet shorter duration of hospital stay is suggested. Jakob et al., in their cross-sectional study that included 2629 who underwent primary LSG, 98.52% of the included patients were discharged after < 24 h from admission and patients’ outcomes in terms of complications rates were not different from the figures available in the literature [11]. So, with medically free patients, who are not expected to have surgical complications, following a fast-track protocol would significantly decrease the overall cost.

#### Postoperative weight loss

In our study, the mean PWL at 6 months postoperatively was 26.41% (SD = 9.79). This figure is close to what we found in the literature. For example, Kavitha Subramaniam et al., in their prospective study that included 57 patients, the PWL was 23.68% (SD = 7.71). In fact, their study included patients who underwent either LSG or LRYGB, and for the patient who underwent LSG, the PWL was 19.84% (SD = 9.79); a figure notably lesser than the one found in KFSH bariatric centre [12]. We used PWS for reporting weight loss in our study because PWL was found to be the parameter least influenced by preoperative BMI. This was the conclusion of a prospective study that included 846 patients. The study, however, included patients who were undergoing Roux-en Y gastric bypass [13].

The relationship between gender and weight loss after bariatric surgery has been assessed in several studies. Some studies showed a better outcome for females [14, 15] and others showed no statistically significant difference [16, 17]. However, we could not find any study showing a better outcome in males. In our study, and for a reason or reasons we could not conclude from the data collected, male patients had a significantly greater PWL compared to females; 30.57% vs. 23.99% (*p *= 0.01). When we looked into the literature, we could not find any study done in SA assessing the impact of gender on post-LSG outcome. In fact, an editorial published in 2016 in the Saudi Journal of Obesity called for an urgent need to set up standards for bariatric surgery in SA, a country where about 15,000 bariatric surgeries are done annually [18]. Such an observation indicates the need for more research on bariatric and metabolic surgery in SA.

#### LSG and type 2 diabetes mellitus

Bariatric surgery does not just reduce body weight. It alleviates the burden of obesity-associated comorbidities [19], and type 2 diabetes is of particular importance, especially to our local context in SA. The World Health Organization has ranked SA in the top ten countries with the highest prevalence of type 2 diabetes in the world [20]. LSG does not only positively affect glucose homeostasis by the metabolic changes that occur secondary to percentage excess weight loss, but also by hormonal changes, such as decreased Ghrelin plasma concentration and increased glucagon-like peptide-1 plasma concentration [21]. Thus, assessing the impact of LSG on type 2 diabetic patients in the contest of SA is highly encouraged in future studies.

### Conclusions

In conclusion, when LSG is performed as a primary bariatric procedure in obese patients, RSG is needless and would unnecessarily increase the overall cost as it makes no difference in detecting leak. Also, a fast-track protocol in admitting patients for LSG and discharging them over 2 days or less would reduce the cost without affecting postoperative outcomes. Finally, the observation of favourable post-LSG outcome in males compared to females in terms of PWL in a Saudi sitting needs to be further investigated in future studies.

## Limitations

The major limitation of this study is the small sample size. This should not influence the conclusions of RSG being cost-ineffective or hospital stay of less than 3 days being cost-effective. On the other hand, it would influence the observation related to the impact of gender on post-LSG PWL. This is why we concluded that more research is needed to assess this point. Another limitation would be the absence of randomization as the study was a records-based cross-sectional study.

## Data Availability

The datasets used and/or analysed during the current study are available from the corresponding author on reasonable request.

## Abbreviations

LSG: Laparoscopic sleeve gastrectomy
LRYGB: Laparoscopic Roux-en Y gastric bypass
RSG: Routine post-sleeve gastrografin
ALOS: Average length of stay
PWL: Percentage weight loss
BMI: Body mass index
SA: Saudi Arabia
KFSH: King Fahd Specialist Hospital
